# Evidence of immune-metabolic imbalance prior to sepsis: a prospective study in the UK Biobank

**DOI:** 10.3389/fnut.2026.1836381

**Published:** 2026-07-16

**Authors:** Xiaoke Liu, Dongqi Zhou, Dazhou Liao, Qiu Chen

**Affiliations:** 1 Department of Endocrine, Hospital of Chengdu University of Traditional Chinese Medicine, Chengdu, Sichuan, China; 2 West China Hospital, Sichuan University, Chengdu, Sichuan, China; 3 Hospital of Chengdu University of Traditional Chinese Medicine, Chengdu, Sichuan, China

**Keywords:** inflammatory susceptibility, metabolic malnutrition, metabolic vulnerability, sepsis, UK Biobank

## Abstract

**Background:**

Sepsis is a severe systemic inflammatory response syndrome, and effective metabolic signatures for predicting its risk are currently lacking. This study seeks to evaluate the associations between inflammatory vulnerability (IVX), metabolic malnutrition (MMX), and metabolic vulnerability (MVX) and the risk of sepsis.

**Methods:**

We conducted a prospective cohort study using data from the United Kingdom Biobank. Cox proportional hazards models were utilized to examine the association between these composite indices and sepsis risk, while restricted cubic spline (RCS) analysis was applied to identify potential nonlinear trends. Subgroup analyses based on medical history, lifestyle factors, and demographic characteristics were performed in fully adjusted models. Sensitivity analyses employed 3-year left truncation and adjusted for CRP and glycated hemoglobin, as well as excluding participants with baseline comorbidities.

**Results:**

In fully adjusted models, a 1-SD increase in MVX corresponded to an 18% higher sepsis risk (HR: 1.18; 95% CI, 1.15–1.20; *p* < 0.01), whereas the highest quartile (Q4) showed a 43% increased risk versus Q1 (HR: 1.43; 95% CI, 1.35–1.52; *p* < 0.01). Positive associations were also observed for IVX (HR per SD: 1.15; 95% CI, 1.13–1.18; *p* < 0.01) and MMX (HR per SD: 1.10; 95% CI, 1.07–1.12; *p* < 0.01). Subgroup demonstrated that MVX was consistently linked to sepsis across various subgroups, with a stronger association than IVX or MMX. Notably, in participants with normal BMI, females, and those with severely decreased eGFR, the risk increased by 23% (HR: 1.23), 26% (HR: 1.26), and 28% (HR: 1.28) per SD increase in MVX, respectively (all *p* < 0.01). RCS analysis confirmed nonlinear dose–response relationships (nonlinear *p* = 0.018), with an inflection point of 38 for MVX; beyond this threshold, sepsis risk increased sharply. Sensitivity analyses supported the aforementioned results.

**Conclusion:**

MVX, IVX, and MMX all demonstrated positive correlations with sepsis risk characterized by distinct nonlinear patterns. The composite index MVX, integrating inflammation and metabolic factors, was slightly more strongly associated with sepsis compared to IVX or MMX.

## 1 Introduction

Sepsis represents a life-threatening clinical syndrome marked by organ dysfunction resulting from an aberrant host response to infection, alongside systemic inflammation and immune dysregulation (1). According to the latest estimates derived from the 2020 global burden of disease data (2), roughly 48.9 million instances of sepsis occur annually worldwide, resulting in about 11 million deaths, which accounts for nearly 20% of all deaths worldwide. Despite continuous advancements in infection control and critical care medicine in recent years, the overall disease burden of sepsis remains persistently high due to the accelerating aging population and the continual rise in patients with chronic underlying conditions. Because of its significant morbidity, mortality, and the pressure it exerts on medical infrastructure (3), sepsis is recognized as a pressing public health emergency. Scoring systems based on clinical parameters, such as the sequential organ failure assessment (SOFA) and quick SOFA, are used to identify sepsis; however, these approaches are hampered by insufficient sensitivity and limitations in risk stratification (4). Moreover, early screening strategies using biomarkers such as C-reactive protein (CRP) and procalcitonin often depend on manifested organ failure and systemic inflammation (5). Given the absence of effective assessment measures, it is clinically imperative to discover reliable tools capable of identifying and stratifying high-risk patients prior to symptom appearance or at the onset of the disease.

Circulating metabolites reflect the comprehensive result of the interaction between endogenous metabolic regulation and exogenous lifestyle factors, and in recent years, they have increasingly been regarded as significant potential biomarkers for assessing the risk of various diseases (6). Throughout the onset and progression of sepsis, the complex interplay of host immune dysregulation, unchecked inflammatory cascades, and bioenergetic reprogramming precipitates profound systemic metabolic perturbations. These pathophysiological shifts underscore the considerable potential of metabolomics for sepsis risk stratification and longitudinal disease monitoring. Prior research has identified notable irregularities in the plasma metabolic profiles of sepsis patients, including lipid metabolism alterations, amino metabolism disorders, and fluctuations in levels of inflammation-related metabolites (7); however, stable metabolic characteristic indicators capable of predicting sepsis occurrence or adverse outcomes have not yet been established. The metabolic vulnerability index (MVX), proposed in recent years, is based on Nuclear Magnetic Resonance (NMR) technology to quantitatively integrate multiple circulating metabolic markers, constructing a comprehensive score that reflects multi-dimensional metabolic imbalance, which can depict the body metabolic vulnerability state more comprehensively compared to single indicators (8). MVX comprises citrate, small high-density lipoprotein (small HDL) particles, acetylated glycoproteins (GlycA), and the branched-chain amino acids (isoleucine, leucine, and valine). Small HDL and GlycA primarily indicate systemic inflammation, whereas citrate and branched-chain amino acids assess metabolic nutritional status and energy metabolism dysfunction (9, 10). It is worth noting that sepsis is frequently characterized by inflammation-driven hypermetabolism, a negative nitrogen balance, and decreased mitochondrial energy production (11, 12). Based on this, it is inferred that elevated MVX may indicate a decline in an individual’s metabolic tolerance to infectious stress, potentially rendering them more susceptible to progressing to sepsis. Nevertheless, there remains a notable absence of systematic population studies regarding the relationship between MVX and the risk of developing sepsis.

As a composite metabolic metric derived from the further integration of IVX, which comprises small HDL and GlycA, and MMX, consisting of the remaining four metabolites, MVX has been shown to be significantly linked to the likelihood of multiple unfavorable health consequences (13), yet its potential value in predicting the risk of sepsis onset remains to be systematically investigated (14). As one of the largest prospective population cohorts globally, the UK Biobank (UKB) provides long-term follow-up clinical outcome data and large-scale metabolomics profiling information, offering a unique opportunity to explore the relationships between metabolic abnormalities and the risk of major diseases (15). Utilizing longitudinal data from the UKB, this study systematically assesses the utility of the MVX and its related indices (IVX, MMX) for predicting sepsis risk and stratifying risk in the general population. Anchored by the recent scaling of UKB metabolomics coverage to approximately 500,000 samples, this study constitutes the first systematic validation of these novel metabolic signatures for sepsis risk identification in a large-scale cohort. Ultimately, this work seeks to furnish novel evidence supporting early risk stratification and the optimization of clinical protocols for sepsis management.

## 2 Materials and methods

### 2.1 UK Biobank study design

**Figure 1:**
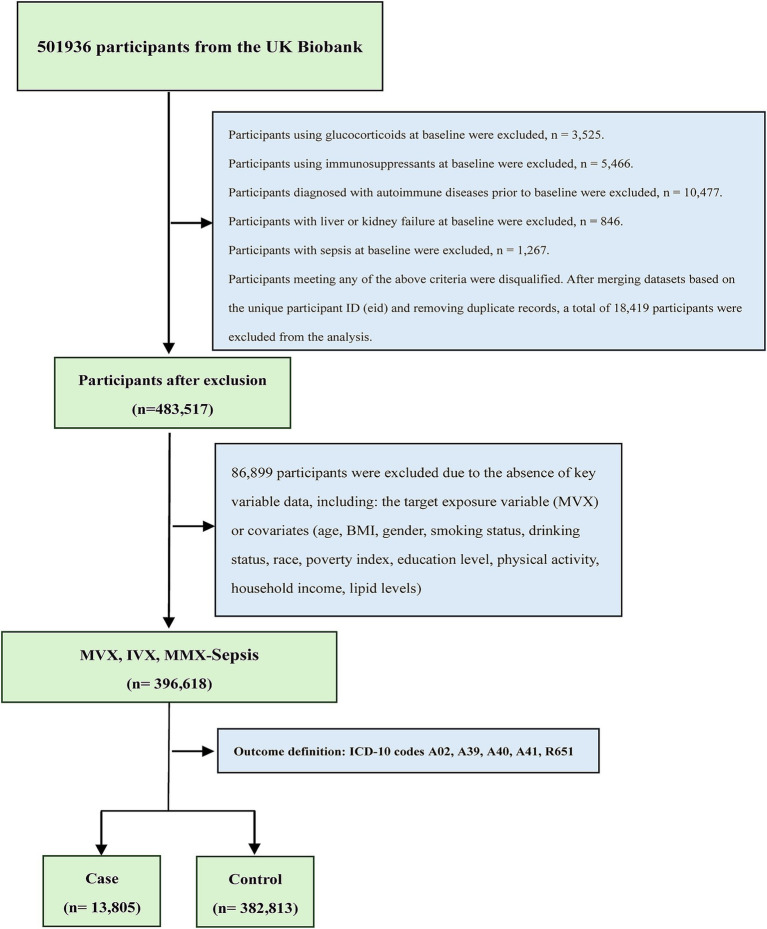
Flowchart of Participant Selection from the UK Biobank for Analysis of MVX, IVX, and MMX-Sepsis Outcomes. This flowchart outlines the process of selecting participants from the UK Biobank for a study examining the relationship between MVX, IVX, MMX, and sepsis outcomes. Initially, 501,936 participants were included. Participants using glucocorticoids or immunosuppressants at baseline, diagnosed with autoimmune diseases prior to baseline, with liver or kidney failure at baseline, or with sepsis at baseline were excluded, resulting in 18,419 exclusions. After merging datasets based on eid and removing duplicate records, 483,517 participants remained. Further exclusions due to missing key variable data reduced the sample size to 396,618 for MVX, IVX, and MMX-sepsis analysis. The outcome was defined using ICD-10 codes A02, A39, A40, A41, R651, resulting in 13,805 cases and 382,813 controls.

This investigation harnessed data from the UK Biobank (UKB), comprising a cohort of more than 500,000 native United Kingdom residents recruited between 2006 and 2010. Comprehensive baseline data were systematically collected, covering sociodemographic characteristics, physical measurements, lifestyle factors, medication history, clinical history, and laboratory biochemical indicators. Participants missing baseline metabolic data and those with sepsis at baseline were eliminated, yielding a final cohort of 396,618 participants. Figure 1 illustrates the detailed participant screening process. This research was funded by UKB project application number 578405. The study was conducted using the UKB resources, which are governed by ethical approval from the North West Multi-centre Research Ethics Committee.

### 2.2 Ascertainment of outcomes

The study outcome was determined by the incidence of sepsis, identified using ICD-10 codes. These codes were used to represent the entire categories, automatically encompassing all relevant subcodes. Specifically, A02 represents salmonella infections, A39 represents meningococcal infection, A40 represents streptococcal sepsis, and A41 represents other sepsis; this approach is consistent with prior literature (16, 17). Given that the Sepsis-3 definition encompasses organ dysfunction resulting from a dysregulated host response to infection (18), we incorporated R65.1 (systemic inflammatory response syndrome of infectious origin with organ failure) as a criterion for identifying organ injury in our analysis. Follow-up commenced at the baseline visit (p53) and ended at the occurrence of the earliest event: outcome diagnosis, death (p40000), loss to follow-up (p191), or March 31, 2023. Participants meeting any of the following baseline criteria were excluded from the study. Baseline use of immunosuppressants or glucocorticoids. A prior diagnosis of autoimmune disease. Severe hepatic or renal insufficiency. A diagnosis of sepsis at baseline. Specifically, baseline glucocorticoid use was ascertained via field ID p20003 and defined to include prednisolone (4 encoded events), dexamethasone, prednisone, and hydrocortisone (1 encoded event each), as well as methylprednisolone (2 encoded events). This definition was restricted exclusively to oral formulations; ophthalmic solutions and topical creams were excluded. Immunosuppressant use was similarly evaluated using field ID p20003, covering 10 distinct immunosuppressive agents—such as cyclophosphamide—comprising a total of 16 encoded events. Baseline autoimmune diseases excluded 11 common immune diseases via 15 coding events. (detailed in the in Supplementary file). Severe hepatic or renal insufficiency was determined based on ICD-10 encoded events, including kidney transplant (Z94.0), dialysis (Z49), end-stage renal disease (N18.5), hepatic failure (K72.0, K72.1, K72.9), and liver transplant (Z94.4).

### 2.3 Metabolic biomarker profiling and MVX calculation

Metabolic biomarker profiles were obtained from EDTA plasma samples collected at the UKB baseline assessment. Nightingale Health performed high-throughput NMR metabolic profiling on theses randomly selected samples. Comprehensive information regarding the experimental and quality control protocols have been previously described (19). The sex-specific calculation method followed previously established protocols (8). MVX was constructed based on six specific metabolites: Citrate (p23473), Isoleucine (p23465), GlycA (p234780), Leucine (p23466), Valine (p23467), and small HDL particles (p23572). The MVX calculation comprises a three-step algorithm: initially, GlycA and small HDL particles are used to determine the IVX; subsequently, the four remaining metabolites are used to calculate the MMX; ultimately, IVX and MMX are aggregated via specific coefficients to yield the final MVX score. MVX scores (range: 1–100) are positively correlated with metabolic fragility, with lower scores suggesting better health and higher scores suggest worse metabolic vulnerability. The specific formula is provided in the Supplementary file.

### 2.4 Ascertainment of covariates

The independent associations of MVX, IVX and MMX with sepsis were evaluated separately. To guarantee the integrity of the results, various of potential confounding factors were corrected in the analysis. The covariates encompassed demographic factors including age (p21003), gender (p31), and ethnicity (p21000), as well as socioeconomic indicators. This category included educational attainment (p6138), household income (p378), physical activity [p884, p894, p904, p914, (20)], and the Townsend Deprivation Index (TDI p22189). Specifically, education was trichotomized as unknown, college or university degree, or other (21). Meanwhile, household income was Subgroup into five groups, spanning from under 18,000 to over 100,000 (22). Lifestyle factors smoking (p20116) and drinking status (p20117) were categorized as current, never, or previous (23). Anthropometric measures included BMI (p21001), classified as obese (BMI ≥ 30.0 kg/m^2^), overweight (BMI between 25.0–29.9 kg/m^2^) and normal (BMI between 18.5–24.9 kg/m^2^), (24). Furthermore, adjustments were made for biochemical markers, including triglycerides (p30870), and cholesterol (p30690). Clinical history adjusted for cancer history (p20001), cardiovascular disease (CVD) history (p20002), chronic liver disease (CLD) history (p20002), and diabetes history (p20002, ICD-10 E11, ICD-9250), chronic respiratory disease (CRD) history (p20002), and AIDS (p20002) (Detailed particulars are provided in the Supplementary file).

### 2.5 Sensitivity analysis

To enhance the reliability of our findings, we conducted a series of stringent sensitivity analyses. First, to mitigate potential bias undetected subclinical cases and reverse causation, we applied a 3-year lag exclusion strategy by eliminating sepsis patients that arose within the initial 3 years of follow-up. Secondly, considering that inflammatory status and glucose metabolism levels may serve as important mediators or confounders in the association between indices such as MVX and sepsis, we incorporated C-reactive protein (CRP) and glycated hemoglobin (HbA1c) as covariates into the fully adjusted Cox proportional hazards regression models, respectively, to verify whether the observed relationships were independent of baseline systemic inflammation and glycemic control. Third, a sensitivity analysis was conducted including only incident sepsis cases defined exclusively by ICD-10 codes A02, A39, A40, and A41. Finally, we conducted an analysis excluding participants with pre-existing CVD, CLD, CRD, diabetes, HIV, or cancer at baseline to re-evaluate the association between MVX and sepsis risk, aiming to verify the generalizability of the results within a general population without these major comorbidities.

### 2.6 Statistical analysis

The FastUKB 3.0 toolkit (25) within the R 3.6.3 environment was used for data extraction, management and preprocessing. Based on the normality test outcomes: uninterrupted data accompanied with a normal distribution are summarized as mean ± SD and compared via Student’s *t*-test or ANOVA; while non-normally distributed metrics are reported as median (IQR) and subjected to the Krusal–Wallis procedures or Mann–Whitney *U*. Regarding categorical variables, we calculated proportions and used the Chi-square test to check for intergroup variations. Statistical significance was established at *p* < 0.05 (two-tailed). We examined the proportionality of hazards via Schoenfeld residuals. Subsequently, multivariate Cox models were employed to quantify the associations between MVX (IVX, MMX) and sepsis, calculating hazard ratios (HRs) and 95% confidence intervals (CIs). Model 1 was adjusted for ethnicity, gender, and age. Model 2 was further adjusted for drinking status, smoking status, TDI, BMI, household income, education level, and physical activity level. Model 3 encompassed all covariates from previous models and additionally adjusted for cancer history, AIDS, CVD, CLD, CRD, diabetes history, blood lipid levels, and eGFR. Based on the Directed Acyclic Graph (DAG), these covariates were identified as potential confounders; therefore, all models sequentially adjusted for these factors to ensure a robust estimation of the independent association between the exposure indices and sepsis. Finally, restricted cubic splines (RCS) were employed to evaluate potential non-linear trends between exposure factors and the risk of sepsis. In accordance with Harrell’s recommendations for survival models, 4 knots were placed at the 5th, 35th, 65th, and 95th percentiles of the MVX distribution. Subgroup analyses were performed by gender, BMI, age, smoking and drinking status, and disease history.

## 3 Results

### 3.1 Baseline characteristics

A total of 396,618 participants were categorized into quartiles by MVX score. Q4 participants were significantly older (median 59.0 vs. 57.0 years) and more frequently female (80.21% vs. 15.50%) compared to Q1 (all *p* < 0.001). Lifestyle factors varied; current drinking and physical activity decreased across quartiles, while smoking increased. Socioeconomic status declined with higher MVX scores. Higher MVX scores correlated with elevated BMI, triglycerides, and cholesterol levels. Additionally, CVD and Cancer prevalence increased across quartiles. Elevated MVX levels were associated with females, older age, lower socioeconomic status, and poorer metabolic and cardiovascular profiles.

### 3.2 Cox proportional hazards model results

**Figure 2:**
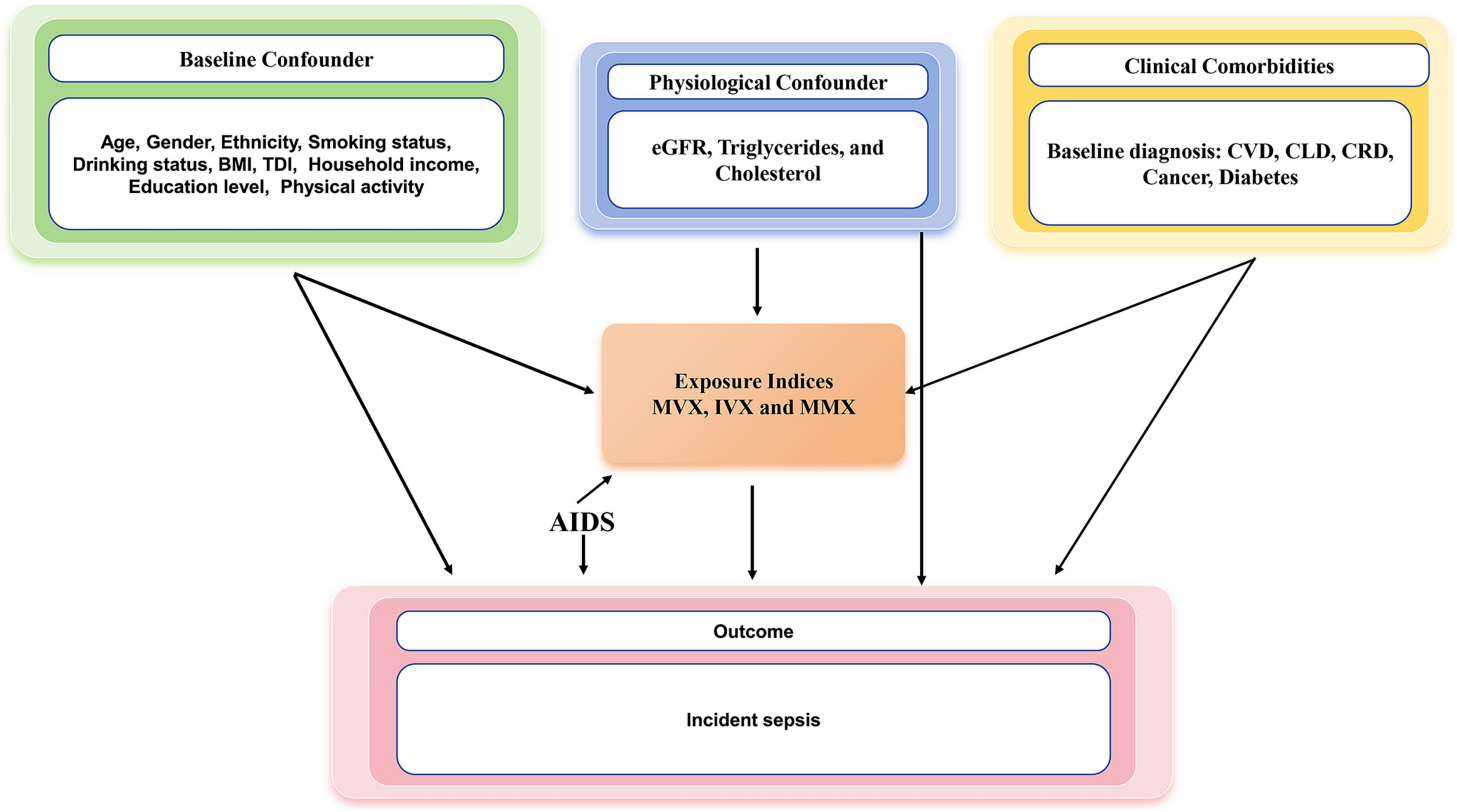
Directed Acyclic Graph (DAG) Depicting the Causal Relationship between Metabolic Vulnerability Indices and Incident Sepsis. This DAG illustrates the causal relationship between exposure indices (MVX, IVX, MMX) and incident sepsis. Baseline confounders (e.g., age, sex, ethnicity, smoking status, alcohol status, BMI, TDI, household income, education level, and physical activity) and physiological confounders (e.g., eGFR, triglycerides, and cholesterol) are linked to both exposure and outcome via solid arrows. AIDS acts as an independent risk factor influencing both the exposure indices and sepsis. Given that the biological mechanisms between MVX and sepsis are not yet fully elucidated, and considering that metabolic vulnerability may induce the development of relevant chronic diseases, this study treats clinical comorbidities (including CVD, CLD, CRD, cancer, and diabetes) as potential confounders and adjusts for them to control for interference in the causal effect estimation; further sensitivity analyses were subsequently conducted.

Figure 2 illustrates the DAG outlining the assumed causal relationships between MVX (IVX and MMX), covariates, and sepsis. Table 1 presents the Cox proportional hazards model results for MVX, IVX, and MMX regarding the risk of sepsis. For MVX (Table 1A), in the comprehensively updated model 3, every one-standard-deviation (per SD) increase in MVX was associated with an 18% increased risk of sepsis (HR = 1.18, 95% CI: 1.15–1.20, *p* < 0.01). Quartile-based analysis revealed that participants in Q4 faced a 43% higher risk of sepsis relative to the reference group Q1 (HR = 1.43, 95% CI: 1.35–1.52, *p* < 0.01). Regarding IVX (Table 1B), in the fully adjusted model 3, per SD increase in IVX was associated with a 15% increased risk of sepsis (HR = 1.15, 95% CI: 1.13–1.18, *p* < 0.01). Participants in Q4 had a 32% higher risk of sepsis compared to Q1 (HR = 1.32, 95% CI: 1.24–1.41, *p* < 0.01). In the case of MMX (Table 1C), in the fully adjusted model 3, every per SD increase in MMX was associated with a 10% increased risk of sepsis (HR = 1.10, 95% CI: 1.07–1.12, *p* < 0.01). Quartile comparisons (Q4 vs. Q1) yielded a HR of 1.20 (95% CI: 1.12–1.28, *p* < 0.01). Detailed results for all models are shown in Table 1. Across all models, the magnitude of association with sepsis was highest for MVX, followed by IVX and then MMX.

**Table 1:** Relationship between exposures and sepsis risk based on the cox proportional hazards model.

	Case *N*	Control *N*	Model 1			Model 2			Model 3		
			HR	95% CI	*p*-value	HR	95% CI	*p*-value	HR	95% CI	*p*-value
A: cox proportional hazards results for MVX on sepsis											
MVX (per SD)	13,805	382,813	1.23	1.21–1.25	<0.01	1.20	1.18–1.23	<0.01	1.18	1.15–1.20	<0.01
MVX Q1	3,512	96,166	1.00	1.00 (Ref)	1.00	1.00	1.00 (Ref)	1.00	1.00	1.00 (Ref)	1.00
MVX Q2	3,314	95,894	1.14	1.09–1.20	<0.01	1.11	1.06–1.17	<0.01	1.11	1.05–1.16	<0.01
MVX Q3	3,024	96,102	1.20	1.14–1.27	<0.01	1.15	1.09–1.21	<0.01	1.14	1.08–1.20	<0.01
MVX Q4	3,955	94,651	1.64	1.56–1.73	<0.01	1.46	1.37–1.54	<0.01	1.43	1.35–1.52	<0.01
B: cox proportional hazards results for IVX on sepsis											
IVX (per SD)	13,805	382,813	1.24	1.21–1.26	<0.01	1.18	1.16–1.21	<0.01	1.15	1.13–1.18	<0.01
IVX Q1	3,924	95,874	1.00	1.00 (Ref)	1.00	1.00	1.00 (Ref)	1.00	1.00	1.00 (Ref)	1.00
IVX Q2	3,218	95,996	1.09	1.04–1.14	<0.01	1.07	1.02–1.13	<0.01	1.05	1.00–1.10	<0.05
IVX Q3	2,812	95,942	1.14	1.08–1.21	<0.01	1.10	1.04–1.16	<0.01	1.06	1.00–1.12	<0.05
IVX Q4	3,851	95,001	1.61	1.53–1.71	<0.01	1.41	1.32–1.50	<0.01	1.32	1.24–1.41	<0.01
C: cox proportional hazards results for MMX on sepsis											
MMX (per SD)	13,805	382,813	1.08	1.06–1.10	<0.01	1.09	1.06–1.11	<0.01	1.10	1.07–1.12	<0.01
MMX Q1	2,808	95,778	1.00	1.00 (Ref)	1.00	1.00	1.00 (Ref)	1.00	1.00	1.00 (Ref)	1.00
MMX Q2	2,961	96,427	0.90	0.86–0.96	<0.01	0.97	0.91–1.02	0.22	0.97	0.92–1.03	0.32
MMX Q3	3,811	96,079	0.97	0.92–1.03	0.40	1.03	0.97–1.10	0.27	1.05	0.98–1.11	0.14
MMX Q4	4,225	94,536	1.13	1.07–1.20	<0.01	1.17	1.11–1.24	<0.01	1.20	1.12–1.28	<0.01

MVX, IVX, and MMX were categorized into quartiles (Q1–Q4), with the lowest quartile (Q1) designated as the reference group. Cox proportional hazards regression models were employed to calculate HRs with their corresponding 95% CIs. Three sequentially adjusted models were constructed: Model 1 adjusted for age, gender, and ethnicity. Model 2 further adjusted for drinking status, smoking status, TDI, BMI, household income, education level, and physical activity. Model 3 additionally considered history of cancer, diabetes, CVD, CLD, CRD, HIV, blood lipids, and eGFR. All covariates were assessed at baseline. *p*-values were two-sided, and statistical significance was defined as *p* < 0.05. Baseline comorbidities included CLD, CVD, CRD, history of cancer, diabetes, and HIV.

HR, Hazard ratio; CI, Confidence interval; TDI, Townsend Deprivation Index; Ref, Reference group; MVX, Metabolic Vulnerability Index; IVX, Inflammation Vulnerability Index; MMX, Metabolic Malnutrition Index; SD, Standard Deviation; CRP, C-Reactive Protein; CVD, Cardiovascular Disease; CLD, Chronic Liver Disease; CRD, Chronic Respiratory Disease; HIV, Human Immunodeficiency Virus.

### 3.3 Subgroup analysis results of sepsis

**Figure 3:**
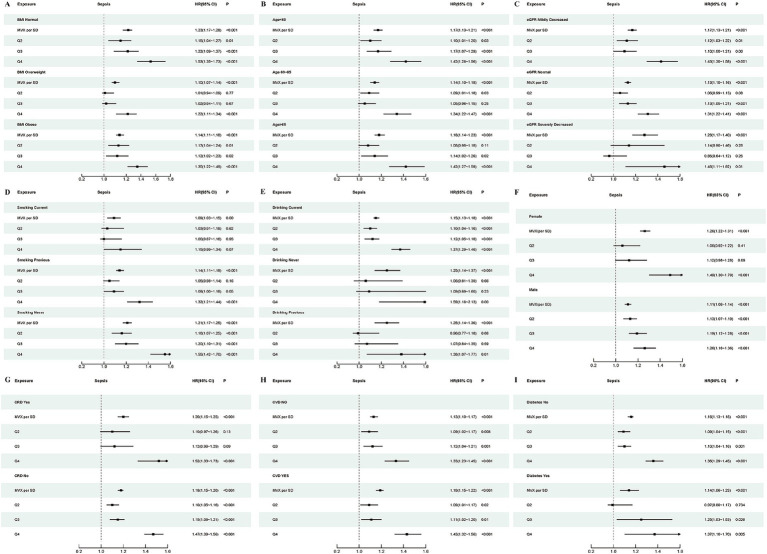
Subgroup Analysis of MVX with Sepsis Across Various Subgroups. **(A–I)** Hazard Ratios (HRs) and their 95% Confidence Intervals (CIs) for the association between MVX and sepsis across various subgroups, including BMI categories (Normal, Overweight, Obese), age groups (<60, 60–65, >65 years), eGFR levels (Mildly Decreased, Normal, Severely Decreased), smoking status (Current, Previous, Never), drinking status (Current, Never, Previous), gender (Female, Male), chronic respiratory disease (CRD Yes/No), cardiovascular disease (CVD Yes/No), and diabetes status (Diabetes Yes/No). Each panel shows the HR per standard deviation (SD) increase in MVX and quartiles (Q1–Q4) of MVX distribution. Statistically significant results are indicated by *p*-values less than 0.05. The figures demonstrate that higher MVX levels are consistently associated with increased HRs for sepsis across all subgroups, with varying degrees of significance depending on the subgroup characteristics.

Subgroup analyses based on the fully adjusted model 3 demonstrated consistent positive associations between MVX and the risk of sepsis across various strata, as illustrated in Figure 3. Regarding BMI stratification, MVX remained significantly linked to sepsis risk across all categories. In individuals with normal BMI, every per SD increase in MVX was associated with a 23% increased risk of sepsis (HR = 1.23, 95% CI: 1.17–1.28, *p* < 0.001). Q4 showed a greater risk compared to Q1 (HR = 1.53, 95% CI: 1.35–1.73, *p* < 0.001). Similarly, for overweight and obese individuals, MVX also exhibited strongly correlated with sepsis risk, with HRs of 1.10 (95% CI: 1.07–1.14, *p* < 0.001) and 1.14 (95% CI: 1.11–1.18, *p* < 0.001), respectively, per SD increase (Figure 3A). Age-Subgroup analysis demonstrated that MVX was significantly associated with sepsis risk across all age groups (Figure 3B), with this association being more pronounced in the ≥65 years group, yielding an HR of 1.18 (95% CI: 1.14–1.23, *p* < 0.001). In terms of eGFR, MVX was significantly associated with sepsis risk across groups with normal eGFR, mild eGFR reduction, and severe eGFR reduction. For patients with mildly reduced eGFR, each one standard deviation increase in MVX was associated with a 17% increase in sepsis risk (HR = 1.17, 95% CI: 1.13–1.21, *p* < 0.001). In the severely reduced eGFR group, this association was even more pronounced, with an HR of 1.28 (95% CI: 1.17–1.40, *p* < 0.001) (Figure 3C). Gender-Subgroup analysis indicated that the MVX-associated risk of sepsis was higher in females than in males. Per one standard deviation increase in MVX, the HR was 1.26 (95% CI: 1.22–1.31, *p* < 0.001) for females and 1.11 (95% CI: 1.08–1.14, *p* < 0.001) for males (Figure 3F). Individuals with CRD had a markedly elevated risk of sepsis linked to MVX in comparison to those without CRD (Figure 3G). Those with CRD had an HR of 1.20 (95% CI: 1.15–1.25, *p* < 0.001) per SD increase in MVX, while those without CRD had an HR of 1.18 (95% CI: 1.15–1.20, *p* < 0.001). MVX shown a substantial correlation with sepsis risk in both patients with and without CVD (Figure 3H). Participants with CVD had an HR of 1.19 (95% CI: 1.15–1.22, *p* < 0.001) per SD increase in MVX, while those without CVD had an HR of 1.13 (95% CI: 1.10–1.17, *p* < 0.001). MVX shown a substantial correlation with sepsis risk in both diabetic and non-diabetic people (Figure 3I). Those with diabetes had an HR of 1.14 (95% CI: 1.06–1.23, *p* < 0.001) per SD increase in MVX, while those without diabetes had an HR of 1.16 (95% CI: 1.13–1.18, *p* < 0.001). MVX was strongly correlated with sepsis irrespective of smoking or drinking status (Figures 3D,E). These data collectively highlight the strength of MVX as a risk factor for sepsis across various demographic and health profiles.

### 3.4 Sensitivity analysis results

The detailed results of the sensitivity analysis are presented in Table 2. In the analysis using 3-year left truncation (Table 2A), the associations for all indices remained significant: per one SD increase in MVX, IVX, and MMX was associated with a 17% (HR: 1.17, 95% CI: 1.15–1.19), 15% (HR: 1.15, 95% CI: 1.12–1.18), and 9% (HR: 1.09, 95% CI: 1.06–1.11) higher risk of sepsis, respectively. After additional adjustment for CRP (Table 2B), the HRs for MVX, IVX, and MMX were 1.14 (95% CI: 1.12–1.16), 1.11 (95% CI: 1.08–1.14), and 1.09 (95% CI: 1.07–1.11), respectively. Similarly, the results remained robust after adjusting for HbA1c (Table 2C): the HR for MVX was 1.17 (95% CI: 1.14–1.19), for IVX was 1.14 (95% CI: 1.11–1.17), and for MMX was 1.09 (95% CI: 1.08–1.11). In Table 2D, regarding sepsis outcomes defined solely by A02, A39, A40, and A41, the associations of the three coefficients with incident sepsis remained robust. Finally, to minimize the influence of pre-existing health conditions, we conducted an analysis excluding participants with baseline comorbidities (Table 2E). In this restricted cohort of 223,927 participants, the positive associations remained consistent. Specifically, in the fully adjusted model, the hazard ratios per 1-SD increase were 1.12 (95% CI: 1.08–1.16) for MVX, 1.10 (95% CI: 1.06–1.14) for IVX, and 1.06 (95% CI: 1.04–1.09) for MMX. Collectively, these sensitivity analyses confirm the stability of the associations between MVX, IVX, MMX, and the risk of sepsis. All *p*-values for the aforementioned results were less than 0.01.

**Table 2:** Sensitivity analysis: relationship between exposures and sepsis risk.

	Case *N*	Control *N*	Model 1			Model 2			Model 3		
			HR	95% CI	*p*-value	HR	95% CI	*p*-value	HR	95% CI	*p*-value
A: sensitivity analysis (3-year left truncation) of sepsis											
MVX (per SD)	12,851	379,580	1.23	1.20–1.25	<0.01	1.19	1.16–1.21	<0.01	1.17	1.15–1.19	<0.01
IVX (per SD)	12,851	379,580	1.23	1.21–1.26	<0.01	1.18	1.15–1.21	<0.01	1.15	1.12–1.18	<0.01
MMX (per SD)	12,851	379,580	1.07	1.05–1.09	<0.01	1.08	1.06–1.10	<0.01	1.09	1.06–1.11	<0.01

	Case *N*	Control *N*	Model 1			Model 2			Model 3 + CRP		
			HR	95% CI	*p*-value	HR	95% CI	*p*-value	HR	95% CI	*p*-value
B: sensitivity analysis (plus CRP adjustment) of sepsis											
MVX (per SD)	13,805	382,813	1.23	1.21–1.25	<0.01	1.20	1.18–1.23	<0.01	1.14	1.12–1.16	<0.01
IVX (per SD)	13,805	382,813	1.24	1.21–1.26	<0.01	1.18	1.16–1.21	<0.01	1.11	1.08–1.14	<0.01
MMX (per SD)	13,805	382,813	1.08	1.06–1.10	<0.01	1.09	1.06–1.11	<0.01	1.09	1.07–1.11	<0.01

	Case *N*	Control *N*	Model 1			Model 2			Model 3 + Hb1Ac		
			HR	95% CI	*p*-value	HR	95% CI	*p*-value	HR	95% CI	*p*-value
C: sensitivity analysis (plus Hb1Ac adjustment) of sepsis											
MVX (per SD)	13,805	382,813	1.23	1.21–1.25	<0.01	1.20	1.18–1.23	<0.01	1.17	1.14–1.19	<0.01
IVX (per SD)	13,805	382,813	1.24	1.21–1.26	<0.01	1.18	1.16–1.21	<0.01	1.14	1.11–1.17	<0.01
MMX (per SD)	13,805	382,813	1.08	1.06–1.10	<0.01	1.09	1.06–1.11	<0.01	1.09	1.08–1.11	<0.01

	Case *N*	Control *N*	Model 1			Model 2			Model 3		
			HR	95%CI	*p*-value	HR	95% CI	*p*-value	HR	95% CI	*p*-value
D: sensitivity analysis (defined solely by A02, A39, A40, and A41) of sepsis											
MVX (per SD)	12,438	384,180	1.22	1.20–1.24	<0.01	1.18	1.15–1.20	<0.01	1.16	1.14–1.18	<0.01
IVX (per SD)	12,438	384,180	1.22	1.20–1.25	<0.01	1.16	1.14–1.19	<0.01	1.14	1.11–1.17	<0.01
MMX (per SD)	12,438	384,180	1.08	1.06–1.10	<0.01	1.08	1.06–1.10	<0.01	1.08	1.06–1.10	<0.01

	Case *N*	Control *N*	Model 1			Model 2			Model 3 – Comorbidities		
			HR	95% CI	*p*-value	HR	95% CI	*p*-value	HR	95% CI	*p*-value
E: sensitivity analysis excluding baseline comorbidities for sepsis											
MVX (per SD)	5,240	218,687	1.16	1.13–1.19	<0.01	1.12	1.08–1.15	<0.01	1.12	1.08–1.16	<0.01
IVX (per SD)	5,240	218,687	1.16	1.12–1.20	<0.01	1.10	1.05–1.14	<0.01	1.10	1.06–1.14	<0.01
MMX (per SD)	5,240	218,687	1.06	1.03–1.09	<0.01	1.06	1.03–1.10	<0.01	1.06	1.04–1.09	<0.01

Cox proportional hazards regression models were employed to calculate HRs with their corresponding 95% CIs. Three sequentially adjusted models were constructed: Model 1 adjusted for age, gender, and ethnicity. Model 2 further adjusted for drinking status, smoking status, TDI, BMI, household income, education level, and physical activity. Model 3 additionally considered history of cancer, diabetes, CVD, CLD, CRD, HIV, blood lipids, and eGFR. All covariates were assessed at baseline. P values were two-sided, and statistical significance was defined as *p* < 0.05. Baseline comorbidities included CLD, CVD, CRD, history of cancer, diabetes, and HIV.

HR: Hazard ratio; CI: Confidence interval; TDI: Townsend Deprivation Index; Ref: Reference group; MVX: Metabolic Vulnerability Index; IVX: Inflammation Vulnerability Index; MMX: Metabolic Malnutrition Index; SD: Standard Deviation; CRP: C-Reactive Protein; CVD: Cardiovascular Disease; CLD: Chronic Liver Disease; CRD: Chronic Respiratory Disease; HIV: Human Immunodeficiency Virus.

### 3.5 RCS analysis results

**Figure 4:**
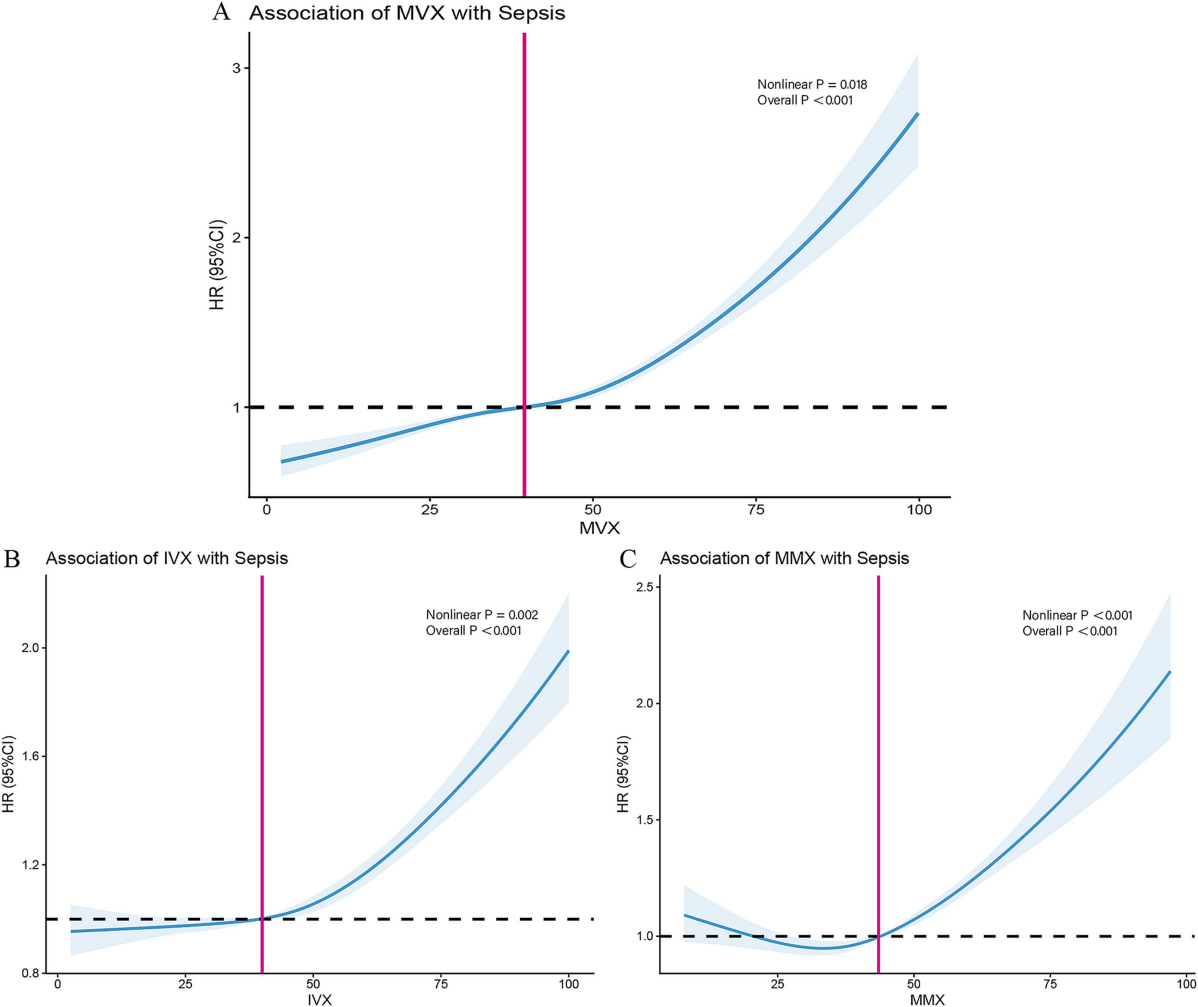
Restricted Cubic Spline Analysis of the Relationship Between MVX, IVX, MMX, and Sepsis. **(A)** Association of MVX with Sepsis. The figure presents a restricted cubic spline (RCS) curve illustrating the nonlinear relationship between MVX and the hazard ratio (HR) for developing sepsis. The blue line represents the estimated HR with its 95% confidence interval (CI) shaded in gray. The dashed black line indicates an HR of 1. An inflection point is marked by a vertical pink line at MVX = 38, indicating a significant turning point where increases in MVX significantly raise the risk of sepsis. **(B)** Association of IVX with Sepsis. This panel shows an RCS curve depicting the nonlinear relationship between IVX and sepsis. Similar to **(A)** the blue line represents the estimated HR with its 95% CI shaded in gray. The dashed black line indicates an HR of 1. An inflection point is marked by a vertical pink line at IVX = 40, indicating a significant turning point where increases in IVX significantly raise the risk of sepsis. **(C)** Association of MMX with Sepsis. This panel presents an RCS curve illustrating the nonlinear relationship between MMX and sepsis. The blue line represents the estimated HR with its 95% CI shaded in gray. The dashed black line indicates an HR of 1. An inflection point is marked by a vertical pink line at MMX = 44, indicating a significant turning point where increases in MMX significantly raise the risk of sepsis.

RCS analysis revealed potential nonlinear relationships between MVX, IVX, and MMX with sepsis (Figures 4A–C). The overall association *p*-values were <0.001 for all three indices, while nonlinear p-values were 0.018, 0.002, and <0.001 for MVX, IVX, and MMX, respectively. Elevated levels of all three indices were significantly associated with the risk of sepsis and exhibited a clear dose–response relationship, in which the risk of sepsis gradually increased as the index levels rose. In the RCS plots, the intersection points of the red and blue lines represent the inflection points. The inflection point for MVX and sepsis is 38, for IVX and sepsis is 40, and for MMX is 44. MVX had the most significant impact on sepsis, as it had the lowest inflection point among the three indices and exhibited the greatest increase in HR, indicating that MVX is an effective and sensitive indicator for assessing the risk of sepsis. Consistent with the findings presented earlier in this study, while single IVX and MMX were significantly associated with sepsis, the MVX, which integrates metabolic malnutrition and inflammatory susceptibility, demonstrated superior predictive power and clinical value.

## 4 Discussion

Metabolic disorders play a significant role in organ dysfunction associated with infections (26); however, previous studies have mostly focused on metabolic changes in critically ill patients after admission (27, 28), and research on metabolic prediction of sepsis risk in the general population remains relatively limited. Additionally, although patients with sepsis exhibit identifiable metabolic abnormalities and subtypes, the consistency of single metabolites across different studies is limited (29, 30), suggesting the need for multi-dimensional integrated composite metrics to provide a more comprehensive assessment. Utilizing UK Biobank metabolomics data, this study systematically investigated the links between MVX, its sub-indices IVX and MMX, and sepsis risk. The results showed that all three metabolic indices were significantly associated with sepsis risk, and the associations remained stable after full multivariate adjustment. Notably, the presence of a distinct dose–response relationship between rising index levels and disease risk implies that metabolic vulnerability likely establishes itself gradually before clinical onset, continuously influencing the host’s response to infection and susceptibility to organ damage.

IVX is characterized by components such as GlycA and small HDL, primarily reflecting low-grade chronic inflammation and inflammation-related lipoprotein alterations. GlycA acts as an integrative signal of acute-phase protein glycosylation, correlating with systemic inflammatory load and diverse pathologies. In UKB NMR spectroscopy studies, the correlation between GlycA and the risk of various illness endpoints, including infection-related outcomes, is particularly prominent, providing population-based evidence for its role as an indicator of inflammatory susceptibility (19, 31). Concurrently, small HDL is not only linked to CVD risk (32) but is also associated with susceptibility to infection and sepsis. While UKB-based data indicate that depleted small HDL levels forecast sepsis risk, Mendelian randomization analyses imply that this relationship may not be strictly causal (33). According to the findings of this study, IVX, composed of the aforementioned two substances, is significantly associated with sepsis, providing new supplementary evidence.

Citrate acts as a key hub in energy metabolism and serves not only as a crucial biomarker for mitochondrial function, but its serum levels also reflect the cellular balance between energy supply and demand (34). The catabolism of BCAAs, (including isoleucine, leucine, and valine) occurs primarily within the mitochondria; consequently, elevated circulating levels of these amino acids are typically viewed as indicators of mitochondrial dysfunction and disordered energy metabolism (35). MMX is composed of metabolites such as citrate and BCAAs, focusing more on signals related to energy metabolism and mitochondrial function. Mitochondrial bioenergetic failure, oxidative stress (OS), and dysregulated substrate utilization are well-established features of sepsis pathogenesis, contributing to adverse outcomes including immunoparalysis and delayed organ recovery (12, 36). Studies on pediatric sepsis indicate that the persistent impairment of mitochondrial respiratory function in peripheral blood mononuclear cells is closely related to delayed organ recovery (36), emphasizing the pathological role of sustained mitochondrial hypo-function in the course of sepsis. In this context, the positive correlation between MMX and sepsis risk suggests that energy metabolism disorders are not merely secondary manifestations following severe illness, but may represent a detectable susceptible phenotype formed prior to clinical events. These findings further reinforce the centrality of metabolic factors in sepsis.

It is noteworthy that the composite index MVX demonstrated a more pronounced magnitude of association with sepsis compared to IVX or MMX in this study. This implies that relying on a single dimension may have limitations in fully capturing the complex etiological landscape of sepsis. Sepsis immunopathology is a systemic outcome driven not by a single pathway, but by the interplay of inflammatory imbalance, metabolic reprogramming, coagulation-endothelial dysfunction, and inter-organ crosstalk. By integrating the two dimensions of inflammation and metabolic nutrition, MVX may reflect a broader aspect of the multi-factorial risk associated with the disease.

Subgroup analysis further provided nuanced insights into the association between MVX and sepsis: this link was more pronounced or possessed greater discriminative power in females, individuals with normal BMI, and younger populations. Previous studies have indicated that there are gender differences in sepsis outcomes, likely involving the multifactorial interplay of sex hormones, immune response intensity, comorbidity profiles, and healthcare accessibility (37). For individuals with normal BMI or those who are younger, traditional clinical risk characteristics may be atypical; therefore, the occult vulnerable state reflected by metabolomics is more likely to precede clinical events, thereby endowing MVX with higher risk indication value in these subgroups. This finding supports the use of metabolic vulnerability to address the limitations of traditional risk stratification, particularly during the preclinical or early stages.

It should be further noted that the significant gender-based differences in MVX levels observed in our baseline characteristics are highly consistent with the baseline findings reported in several prior MVX studies based on the UK Biobank cohort (13, 38–41). Given that a large proportion of females in the UKB are in the peri- or post-menopausal stage, this distribution pattern can be further explained by female-specific physiological and pathological characteristics. Accumulating evidence indicates that the drastic hormonal fluctuations during the menopausal transition lead to the accumulation of multiple cardiometabolic risk factors, rapidly exacerbating systemic chronic inflammation and causing HDL dysfunction (42, 43). Given that MVX inherently incorporates the dimension of inflammatory vulnerability, these intense physiological changes in women likely serve as a key driver elevating MVX levels. On the other hand, compared to males, the female metabolic system exhibits greater instability. Evidence supporting this theory shows that at any given consistent BMI level, females are more prone to metabolic disorders such as insulin resistance than males (44). More importantly, recent authoritative studies indicate that women undergo a degree of post-menopausal immune remodeling that is twice that of men (45). This implies that, during the aging process, female immune homeostasis is more susceptible to disruption, leading to a higher risk of immune-related susceptibility (45). Consequently, the elevated MVX levels or stronger associations observed in females may reflect the index’s sensitivity in capturing the accelerated deterioration of metabolic and immunological health driven by estrogen deficiency.

The observed differences in the association between MVX and sepsis across eGFR subgroups warrant careful interpretation. While this variation may partly stem from reduced metabolite clearance capacity due to declined renal function, we contend that it also reflects a more profound pathological interplay. Renal dysfunction itself exacerbates metabolic-immune homeostatic imbalance (46, 47), thereby amplifying the impact of the intrinsic vulnerability represented by MVX on the risk of sepsis. Specifically, the normal operation of the kidneys, such as reabsorption, is highly dependent on energy supply (48). Mitochondrial dysfunction not only directly causes kidney injury but also triggers OS. This OS further damages mitochondrial structures, thereby exacerbating the vicious cycle between renal impairment and mitochondrial dysfunction (49). Moreover, clinical evidence indicates that patients with kidney disease exhibit significantly elevated systemic inflammation indices (50), and those on dialysis face a substantially increased risk of sepsis (51). In particular, given that existing studies have confirmed a prospective association between MVX and the development and progression of chronic kidney disease (39), this suggests that metabolic vulnerability itself serves as a critical driver or a pathological marker of renal function impairment. In this context, combined with our observed associations, elevated MVX may synergize with impaired eGFR in patients with renal insufficiency to increase the risk of sepsis.

The findings from our sensitivity analyses underscore the robustness and stability of the identified relationships between MVX, IVX, and MMX and the risk of sepsis. First, the persistence of significant associations following a 3-year left truncation mitigates concerns regarding reverse causation. Second, the associations remained evident even after additional adjustment for CRP and HbA1c, indicating that the predictive value of MVX is not merely a reflection of systemic inflammation or chronic hyperglycemia alone, but rather captures a distinct pathological susceptibility related to multi-system metabolic dysfunction. Furthermore, the consistency of results in the restricted cohort excluding participants with baseline comorbidities implies that these metabolic indices serve as early risk indicators independent of established chronic diseases. Collectively, these validation steps confirm that metabolic vulnerability represents a fundamental and independent risk factor for sepsis, maintaining its prognostic utility across various clinical scenarios and adjustment strata.

The identification of the MVX ≥ 38 inflection point provides critical evidence for clinical stratification. This threshold signifies that metabolic vulnerability transitions into a distinct pathological state at this level, where the cumulative burden of inflammation and mitochondrial dysfunction exceeds physiological adaptive capacity. Prior to this inflection point, the body likely relies on adaptive regulatory mechanisms to maintain homeostasis; analogous examples include the early-stage enhancement of insulin secretion to counteract insulin resistance (52) or the safe sequestration of excess lipids in subcutaneous adipose tissue (53, 54), both of which represent the body’s inherent capacity for adaptation. Similarly, within the context of mitochondrial dynamics, functional mitochondria initially maintain stability and integrity by regulating the dynamic balance between fusion and fission (55). Conversely, once the threshold of dysregulation is crossed, it appears to precipitate irreversible ROS-mediated damage, vascular endothelial injury, and cellular dysfunction (12, 55), which are key pathogenic hallmarks observed in sepsis (56–58). Clinically, the defined threshold of 38, derived from our analyses in the UK Biobank, serves as a potential screening tool to identify individuals at high risk for sepsis who might otherwise be overlooked by traditional risk factors. These findings support a phased prevention strategy rather than viewing metabolic risk as a linear continuum: individuals below this threshold may maintain metabolic homeostasis through lifestyle modifications, whereas those approaching or exceeding it should be prioritized for more intensive monitoring, early intervention, or further immunometabolic assessment.

This study has several limitations. First, metabolites were mostly measured at a single baseline time point, making it difficult to characterize metabolic trajectories over time; this may result in regression dilution bias and an underestimating of the actual effects. Second, the UKB population is predominantly of European ancestry; thus, generalizability to other populations should be approached with caution, and the transferability of the composition and weights of MVX across different populations requires further validation. Third, due to data access limitations, we were unable to adjust for potential confounders such as baseline medications (e.g., statins, metformin, and ACEi/ARBs) or to perform formal interaction tests (e.g., MVX × eGFR) to statistically assess effect modification. Consequently, while we observed subgroup differences, we cannot rule out residual confounding or fully quantify the statistical synergy between these factors based on the current models. Finally, the lack of further gender-stratified analysis in our study may also introduce potential bias to the observed results.

## 5 Conclusion

This prospective cohort study utilizing UKB data suggests that MVX, IVX, and MMX are all significant indicators of sepsis risk, with MVX exhibiting the most pronounced association. The findings reveal a nonlinear dose–response relationship, indicating that sepsis risk increases sharply when MVX exceeds a specific threshold (38). Furthermore, the association of with sepsis remained consistent across diverse subgroups, particularly among females, individuals with normal BMI, and those with impaired kidney function, suggesting its potential as an integrated metabolic-inflammatory signature for sepsis risk assessment.

## Data Availability

The data analyzed in this study was obtained from UK Biobank, the following licenses/restrictions apply: the dataset is accessible to qualified scientists engaged in health-related inquiries that serve the public good. Researchers interested in acquiring these materials should consult the official guidelines regarding the access protocol at: http://www.ukbiobank.ac.uk/register-apply.
