# Supplementary material for: Evidence of immune-metabolic imbalance prior to sepsis: a prospective study in the UK Biobank
Source: Front Nutr. 2026 Jul 16;13:1836381. doi: 10.3389/fnut.2026.1836381 (PMC13421901; doi:10.3389/fnut.2026.1836381)
Supplement: Supplementary file 1 [file Table_1.docx]

**Supplementary Methods**

**1.Details of Baseline Exclusion**

At baseline, we excluded participants who were using hormones or immunosuppressants. Specifically, for the hormone category (p20003), individuals were excluded if they reported the use of Prednisolone (covering 5 coding events: 1140874930, 1140874976, 1140883026, 1141157402), Prednisone (1140868364), Methylprednisolone (covering 2 coding events: 1140874976, 1140883026), Dexamethasone (covering 5 coding events: 1140874816), or Hydrocortisone (1140875668). Regarding immunosuppressants, we excluded those using Methotrexate (covering 2 coding events: 1140869848, 1140910036), Azathioprine (1140869930, 1140909864), Cyclophosphamide (1140869604), Mycophenolate (1140925978), Leflunomide (1141166294, 1141166302, 1141166304, 1141166306), Ciclosporin (1140909844, 1141181020), Tacrolimus (1140911642, 1141179842), Hydroxychloroquine (1140884308), or Sulfasalazine (1140909702).

**2.Covariates assessment**

Cardiovascular disease (CVD) and chronic liver disease (CLD) status were determined using non-cancer diagnosis records (P20002) and the year of first non-cancer diagnosis (P20008). A history of CVD was defined as yes if the participant had any of the ICD-10 codes—specifically 1067, 1068, 1078, 1079, 1080, 1081, 1082, 1086, 1087, 1088, 1093, 1094, 1425, 1426, 1065, 1479, 1483, 1484, 1485, 1486, 1487, 1488, 1489, 1490, 1491, 1492, 1583, 1584, 1585, 1586, 1587, 1588, 1589, 1590, 1591, or 1592—recorded before baseline; otherwise, it was classified as no. Similarly, a positive history of CLD was assigned if any of the codes 1155, 1156, 1157, 1158, 1506, 1507, 1508, 1578, 1579, 1580, 1581, 1582, or 1604 were present in the pre-baseline records, and no otherwise. Furthermore, Cancer history (Cancer history) was determined based on cancer diagnosis reports (P20001) and the year of first cancer diagnosis (P20006). If a participant had any record of cancer diagnosis prior to the baseline, the variable was coded as yes; conversely, it was coded as no. Additionally, chronic respiratory disease (CRD) status was identified using non-cancer diagnosis records (P20002) and the year of first non-cancer diagnosis (P20008). A history of CRD was defined as yes if the participant had any of the codes—specifically 1111, 1112, 1113, 1472, 1114, 1115, 1121, 1122, or 1496—recorded before baseline; otherwise, it was classified as no.

**3.MVX calculation**

Values for each metabolic biomarkers were winsorized at the 1st and 99th percentile. Multi-marker scores were normalized using min-max scaling to a range of 1 to 100 before analysis. The sex-specific equations are provided below, with reference to the CATHGEN biorepository[1].

R script:

df %>% mutate(

IVX = case_when(

sex == "F" ~ 9 - (GlycA * 0.000187) - (sHDL * 0.3585) + (GlycA * sHDL * 0.000348),

sex == "M" ~ 9 - (GlycA * 0.00437) - (sHDL * 0.52307) + (GlycA * sHDL * 0.000817)

),

MMX = case_when(

sex == "F" ~

((4 - (Leucine * 0.03142) + (Leucine^2 * 0.0000893)) * 0.353) +

((7 - (Valine * 0.03362) + (Valine^2 * 0.0000689)) * 0.684) +

(Isoleucine * 0.00332) +

((1 - (Citrate * 0.0072) + (Citrate^2 * 0.0000573)) * 0.7135),

sex == "M" ~

((4 - (Leucine * 0.01594) + (Leucine^2 * 0.0000291)) * 1.076) +

((7 - (Valine * 0.0239) + (Valine^2 * 0.00005)) * 0.414) +

(Isoleucine * 0.01265) +

((1 + (Citrate * 0.00906) - (Citrate^2 * 0.0000126)) * 0.5881)

),

MVX = case_when(

sex == "F" ~

(IVX * 2.27278) + (log(MMX) * 12.13511) - (IVX * log(MMX) * 1.09312),

sex == "M" ~

(IVX * 3.54601) + (log(MMX) * 14.41428) - (IVX * log(MMX) * 1.43438)

)

)

For the female cohort:

The IVX calculation formula is: 9 + (GlycA × -0.000187) + (sHDL × -0.3585) + ((GlycA × sHDL) × 0.000348).

The MMX calculation comprises two weighted components, as per the following formula:

MMX = ((4 + (Leu × -0.03142) + (Leu²) × 0.0000893) × 0.353) + ((7 + (Val × -0.03362) + (Val²) × 0.0000689) × 0.684) + (Ile × 0.00332) + ((1 + (Citr × -0.0072) + (Citr²) × 0.0000573)) × 0.7135.

The calculation logic for MVX is: (IVX × 2.27278) + (ln(MMX) × 12.13511) + (IVX × ln(MMX)) × -1.09312.

For the male cohort:

The IVX formula is: 9 + (GlycA × -0.00437) + (sHDL × -0.52307) + ((GlycA × sHDL) × 0.000817).

The MMX formula is adjusted to:

MMX = ((4 + (Leu × -0.01594) + (Leu²) × 0.0000291) × 1.076) + ((7 + (Val × -0.0239) + (Val²) × 0.00005) × 0.414) + (Ile × 0.01265) + ((1 + (Citr × 0.00906) + (Citr²) × -0.0000126)) × 0.5881.

The formula for calculating MVX is: (IVX × 3.54601) + (ln(MMX) × 14.41428) + (IVX × ln(MMX)) × -1.43438.

**References**

1. Otvos, J.D., et al., Multimarkers of metabolic malnutrition and inflammation and their association with mortality risk in cardiac catheterisation patients: a prospective, longitudinal, observational, cohort study. Lancet Healthy Longev, 2023. 4(2): p. e72-e82.
